# Prevalence, Outcomes and Healthcare Costs of Postoperative ARDS Compared with Medical ARDS

**DOI:** 10.3390/jcm14145125

**Published:** 2025-07-18

**Authors:** Miguel Bardají-Carrillo, Rocío López-Herrero, Mario S. Espinoza-Fernández, Lucía Alonso-Villalobos, Rosa Cobo-Zubia, Rosa Prieto-Utrera, Irene Arroyo-Hernantes, Esther Gómez-Sánchez, Luigi Camporota, Jesús Villar, Eduardo Tamayo

**Affiliations:** 1 BioCritic, Group for Biomedical Research in Critical Care Medicine, 47003 Valladolid, Spain; mbardaji@saludcastillayleon.es (M.B.-C.); msespinoza@saludcastillayleon.es (M.S.E.-F.); egonmezs@saludcastillayleon.es (E.G.-S.); etamayog@saludcastillayleon.es (E.T.); 2 Anesthesiology and Critical Care, Clinical University Hospital of Valladolid (HCUV), 47003 Valladolid, Spain; 3 Department of Surgery, University of Valladolid, 47003 Valladolid, Spain; 4 CIBER de Enfermedades Infecciosas (CIBERINFEC), Instituto de Salud Carlos III, Avenida Monforte de Lemos 5, 28029 Madrid, Spain; 5 Department of Research and Innovation, Clinical University Hospital of Valladolid (HCUV), SACYL/IECSCYL, 47003 Valladolid, Spain; 6 Department of Adult Critical Care, Guy’s and St Thomas’ NHS Foundation Trust, Westminster Bridge Road, London SE1 7EH, UK; 7 Centre for Human and Applied Physiological Sciences, King’s College London Strand, London WC2R 2LS, UK; 8 CIBER de Enfermedades Respiratorias, Instituto de Salud Carlos III, Avenida Monforte de Lemos 5, 28029 Madrid, Spain; jesus.villar54@gmail.com; 9 Research Unit, Hospital Universitario Dr. Negrín, Fundación Canaria Instituto de Investigación Sanitaria de Canarias, Barranco de la Ballena s/n, 35019 Las Palmas de Gran Canaria, Spain; 10 Li Ka Shing Knowledge Institute, St. Michael’s Hospital, 209 Victoria St, Toronto, ON M5B 1T8, Canada; 11 Faculty of Health Sciences, Universidad del Atlántico Medio, Carretera de Quilmes 37, Tafira Baja, 35017 Las Palmas, Spain

**Keywords:** acute respiratory distress syndrome, ARDS, postoperative ARDS, prevalence, mortality, healthcare cost

## Abstract

**Background/Objectives**: Postoperative acute respiratory distress syndrome (ARDS) is a recognized complication with reported prevalence rates of up to 20% and highly variable mortality. However, there is limited published evidence comparing the outcomes of postoperative ARDS with those of medical ARDS. We aimed to evaluate the prevalence, hospital mortality, and healthcare costs of postoperative ARDS in Spain between 2000 and 2022 and to compare them with those of medical ARDS. **Methods**: We performed a nationwide, registry-based study of all hospitalizations for postoperative ARDS in Spain between 1 January 2000 and 31 December 2022 using the Minimum Basic Data Set (MBDS) Registry. **Results**: We identified a total of 93,192 ARDS patients, of which 40,601 had postoperative ARDS. The postoperative ARDS prevalence varied between 0.05 and 0.22%, accounting for 45–50% of total ARDS cases recorded during the study period. Hospital mortality was lower in postoperative ARDS compared with medical ARDS during the first phase (2000–2015) (47.0% vs. 49.9%, *p* < 0.001) and converged during the second phase (2017–2022) (42.7% vs. 43.2%, *p* = 0.413). Postoperative ARDS was associated with a longer hospital stay and 1.5 times higher healthcare costs compared with medical ARDS. During the COVID-19 pandemic, mortality rates declined but costs peaked in both groups. The incidence of digestive tract infection was higher in postoperative ARDS. **Conclusions**: The prevalence of postoperative ARDS remained stable, except during the COVID-19 pandemic, and its hospital mortality declined and equalized with that of medical ARDS. However, the costs associated with postoperative ARDS remained significantly higher.

## 1. Introduction

Postoperative acute respiratory failure (PARF) and postoperative pulmonary complications (PPCs) are major contributors to morbidity and mortality and are associated with prolonged length of hospital stay (LoS) and increased healthcare costs [1,2]. A leading cause of PARF and PPCs is postoperative acute respiratory distress syndrome (ARDS) [3]. Given that over 230 million surgical procedures are performed annually worldwide [2,4], the impact of postoperative ARDS may be underappreciated.

The risk of developing postoperative ARDS varies depending on the type of surgical procedure. Cardiac, thoracic, vascular, and trauma surgeries carry a higher risk [5]. The reported prevalence ranges from 0.2% to 20% [6]. Emergency surgery is a major risk factor, increasing the likelihood of postoperative ARDS ninefold [5]. The mortality rates associated with postoperative ARDS vary widely [6,7] and appear largely independent of preoperative comorbidities [5].

Giannakoulis et al. [7] reported that postoperative ARDS is associated with lower mortality than medical ARDS. While advancements in intraoperative management and implementation of optimal ventilatory and fluid strategies might contribute to improved outcomes, it is plausible that the prevalence of postoperative ARDS has declined over time [7,8]. There is currently limited evidence comparing ARDS in medical vs. postsurgical patients, and comprehensive data on the epidemiology of postoperative ARDS remain scarce. Moreover, the impact of COVID-19 on the epidemiology of ARDS [9] and its specific effect on postoperative ARDS have not been fully evaluated.

We aimed to evaluate the prevalence, hospital mortality, and healthcare costs of postoperative ARDS in Spain between 2000 and 2022 and to compare them with those of medical ARDS over the same time period. We also evaluated temporal trends, including the potential impact of the COVID-19 pandemic on the outcomes, and examined the clinical features associated with postoperative ARDS.

## 2. Materials and Methods

### 2.1. Study Design and Data Source

We performed a nationwide registry-based retrospective study of all ARDS hospitalizations in Spain between 1 January 2000 and 31 December 2022, taking into account that before 2016 only public hospitals were included in the Minimum Basic Data Set (MBDS), while from 2016 onwards both public and private hospitals were included. We analyzed only ARDS patients who received invasive MV [10]. The year 2016 was excluded from the analysis because the Spanish Ministry of Health was unable to provide data for that year due to problems with the implementation of a new model for MBDS [11]. Afterwards, we divided ARDS patients on postoperative ARDS and medical ARDS based on the MBDS data.

Clinical and administrative data were sourced from hospital records in the MBDS of the National Surveillance System for Hospital Data in Spain, provided by the Ministry of Health and published annually with a two-year delay. The MBDS is a clinical and administrative database that includes clinical information recorded at the time of hospital discharge, with an estimated coverage of 99.5% [11,12], providing a robust and consistent foundation for the data. The MBDS includes up to 20 diagnoses, indicating whether the diagnosis was present on admission, as well as 20 therapeutic procedures performed during the hospital stay. The MBDS provides encrypted patient identification numbers, along with information on sex, date of birth, dates of hospital admission and discharge, medical institutions providing the services, diagnosis and procedure codes, and the outcome at discharge, according to the International Classification of Diseases 9th Revision, Clinical Modification (ICD-9-CM) [13] or the International Classification of Diseases 10th Revision, Clinical Modification (ICM-10-CM) [14] depending on the period of the study. The number of surgical interventions performed in Spain from 2010 to 2022, used to calculate the prevalence of postoperative ARDS, was obtained from the Spanish Ministry of Health [15,16], with no data available from previous years.

Data were handled in strict accordance with Spanish confidentiality regulations. This study was approved by the Ethics Committee for Clinical Research of Valladolid East Health Area (#PI-24-399-C). Given the anonymity and nature of the collected data, informed consent was waived.

### 2.2. Study Variables

We included all hospitalized patients in public and private hospitals in Spain with a diagnosis of respiratory distress syndrome (ICD-9-CM codes 518.82 and 518.5 and ICD-10-CM codes J80.*). For the purpose of this study, we also selected the need for mechanical ventilation (MV) (ICD-9 codes 96.70, 96.71, 96.72, and 96.04 and ICD-10 codes 5A1935Z, 5A1945Z, and 5A1955Z). Postoperative patients were defined if they had a surgical intervention date attached. We applied no exclusion criteria. Sepsis was defined using the codes adapted by MacLaren et al. [17], Esper et al. [18], Dombrovskiy et al. [19], and Bateman et al. [20] (Supplementary Tables S1 and S2). The source of infection was defined by codes adapted by Esper et al. [18] and Wang et al. [21] (Supplementary Tables S3 and S4), as well as by organ dysfunction (Supplementary Tables S5 and S6) according to sepsis criteria by Angus [22], adapted by Shen et al. [23] and Bateman et al. [20]. The Charlson Comorbidity Index was calculated from data extracted from the MBDS (Supplementary Tables S7 and S8). All codes were updated to ICD-10-CM by our group [24].

The collected data included age, sex, comorbidities (diabetes, hypertension, heart disease, chronic renal disease, respiratory disease, and neurological disease), site of infection, LoS, and hospital mortality. The study was divided into two periods, corresponding to the change in the ARDS codification due to the transition from ICD-9-CM to ICD-10-CM between 2015 and 2016. The primary outcomes were postoperative ARDS prevalence, hospital mortality, and mean healthcare costs per postoperative ARDS patient. Secondary outcomes included LoS, site of infection, comorbidities, and effects of COVID-19 on epidemiology of postoperative ARDS.

### 2.3. Statistical Analysis

The postoperative ARDS prevalence was calculated as the proportion of patients who developed ARDS requiring MV after undergoing surgery (excluding day-case surgery, as obtained from the MBDS) relative to the total number of patients who underwent surgery (excluding day-case surgery). Hospital mortality was calculated as the proportion of overall hospital deaths in ARDS patients. Differences among groups were assessed using the chi-square test for categorical variables and the ANOVA test for continuous variables normally distributed, as well as the Kruskal–Wallis test for continuous variables not normally distributed. We used the Kolmogorov–Smirnov test to assess the normality of data. Categorical variables are expressed in percentages, and continuous variables expressed as the mean ± standard deviation (SD). The LoS was obtained as the difference in days between the date of hospital admission and the date of discharge (in survivors) or death (in non-survivors). Day of hospital admission was considered day 0. Discharge on the same day was considered as a 1-day stay. Costs were calculated using diagnosis-related groups (DRGs), which represents a medical–economic entity concerning a set of diseases requiring analogous management resources. The DRG data were extracted from the MBDS. A univariate logistic regression adjusted for age and sex was conducted with postoperative ARDS as the outcome (Supplementary Tables S9 and S10). Variables with *p* < 0.05 were included in the multivariate analysis. All analyses were performed using the R statistical package, version 4.3.2 [25]. All tests conducted were two-tailed, and *p*-values <0.05 were considered significant.

## 3. Results

### 3.1. Patients’ Characteristics

Table 1 and Table 2 show the characteristics of patients admitted in the period 2000–2015 and in the period 2017–2022, respectively. A total of 93,192 patients were diagnosed with ARDS between 2000 and 2022. Of those, 68,213 between 2000 and 2015 (36,393 medical ARDS and 31,820 postoperative ARDS) and 24,979 between 2017 and 2022 (16,198 medical ARDS and 8781 postoperative ARDS). Surgical patients were older than the medical ARDS patients during the first study period (60.3 ± 19.7 vs. 56.9 ± 20.8 years, *p* < 0.001). No age difference was observed in the second period. In both periods, ARDS was more common in males. The Charlson Comorbidity Index was significantly higher in surgical compared with medical patients in both periods (0.92 ± 0.98 vs. 0.73 ± 0.86, *p* < 0.001; 1.25 ± 1.76 vs. 1.01 ± 1.41, *p* < 0.001). The mean LoS was 12–13 days longer in postoperative ARDS.

Gastrointestinal infections were the most frequent cause of postoperative complications in both study periods [9.9% (n = 3681) vs. 18.1% (n = 5753), *p* < 0.001; 1.6% (n = 253) vs. 12.3% (n = 1080), *p* < 0.001], whereas respiratory infections were most frequent in medical patients. Obesity was more prevalent in medical ARDS during the second period [20.7% (n = 3354) vs. 15.0% (n = 1314), *p* < 0.001]. Sepsis was more frequent in postoperative ARDS patients during the second period [48.88% (n = 7917) vs. 59.06% (n = 5186), *p* < 0.001]. Extracorporeal membrane oxygenation (ECMO) was used more frequently in postoperative ARDS patients than in medical ARDS (Table 1 and Table 2).

**Figure 1:**
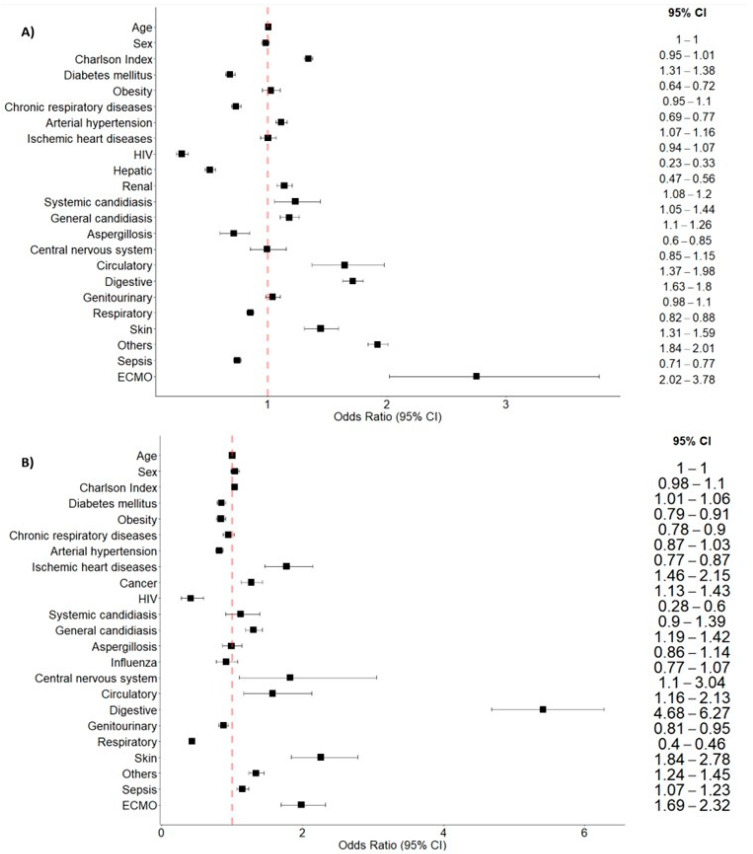
Box plot of the multivariate analysis of the factors associated with postoperative acute respiratory distress syndrome (ARDS): (**A**) multivariate analysis of the first period of the study (2000–2015); (**B**) multivariate analysis of the second period of the study (2017–2022).

In the multivariate analysis of the first study period (Figure 1A), gastrointestinal infection (OR 1.7, 95%CI 1.6–1.8, *p* < 0.001) emerged as the strongest factor associated with postoperative ARDS compared with medical ARDS. During the second period of the study (Figure 1B), gastrointestinal infection (OR 5.4, 95%CI 4.7–6.3, *p* < 0.001) was associated with postoperative ARDS, together with skin infection (OR 2.3, 95%CI 1.8–2.8, *p* < 0.001). A higher Charlson Index was independently associated with postoperative ARDS in both study periods (OR 1.3, 95%CI 1.3–1.4, *p* < 0.001; OR 1.0, 95%CI 1.0–1.1, *p* = 0.006), as well as circulatory source of infection (OR 1.6, 95%CI 1.4–2.0, *p* < 0.001; OR 1.6, 95%CI 1.2–2.1, *p* = 0.003).

### 3.2. Prevalence

**Figure 2:**
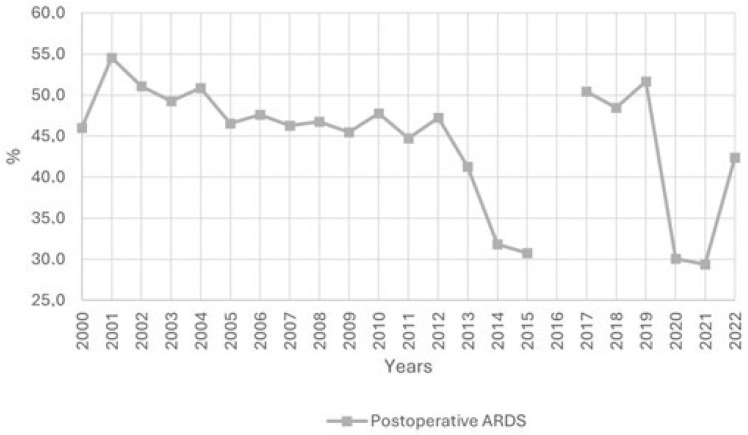
Evolution of the percentage of ARDS patients that are postoperative ARDS from total ARDS patients from 2000 to 2022.

**Figure 3:**
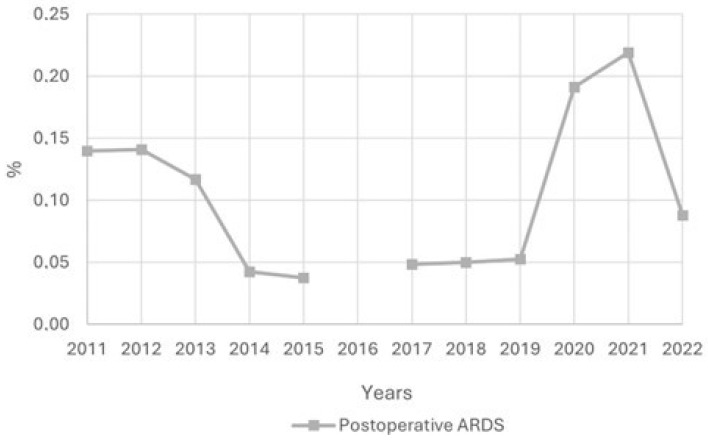
Evolution of postoperative ARDS prevalence (percentage from total surgeries) between 2011 and 2022.

Figure 2 shows the proportion of patients with postoperative ARDS from 2000 to 2022. During the first study period, postoperative ARDS accounted for about 45–50% of all ARDS cases, with the exception of 2014 and 2015, during which the proportion dropped to 31.8% and 30.7%, respectively. In the second period, postoperative ARDS represented about 50% of all ARDS cases. This rate declined with the onset of the COVID-19 pandemic, reaching a low of 29.4% in 2021. As the pandemic receded in 2022, the proportion of postoperative ARDS patients rose to 42.4%. The trend in postoperative ARDS prevalence is shown in Figure 3. The prevalence of postoperative ARDS varied in Spain between 0.05% and 0.22%, peaking in 2021.

### 3.3. Hospital Mortality

**Figure 4:**
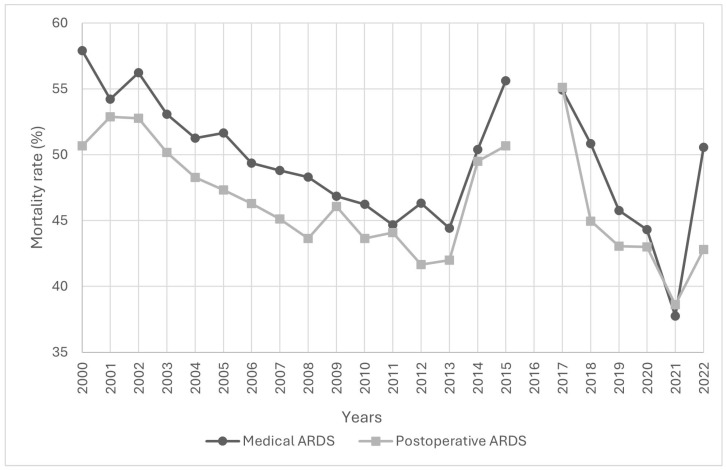
Evolution of medical ARDS and postoperative ARDS hospital mortality between 2000 and 2022 in Spain.

The trend in hospital mortality is shown in Figure 4. As reported in Table 1, mortality was higher in medical ARDS than in postoperative ARDS during the first period (49.9% (n = 18,168) vs. 47.0% (n = 14,943), *p* < 0.001). No difference in mortality was observed between the two groups during the second period (43.2% (n = 7004) vs. 42.7% (n = 3749), *p* = 0.413). Hospital mortality declined over time. In postoperative ARDS patients, mortality decreased from 50.7% in 2000 to 42.8% in 2022, while in medical ARDS patients, it declined from 57.9% in 2000 to 50.6% in 2022. Postoperative ARDS mortality was higher than medical ARDS mortality in 2017 and 2021, but these differences were not significant.

### 3.4. Mean Costs per ARDS Patient

**Figure 5:**
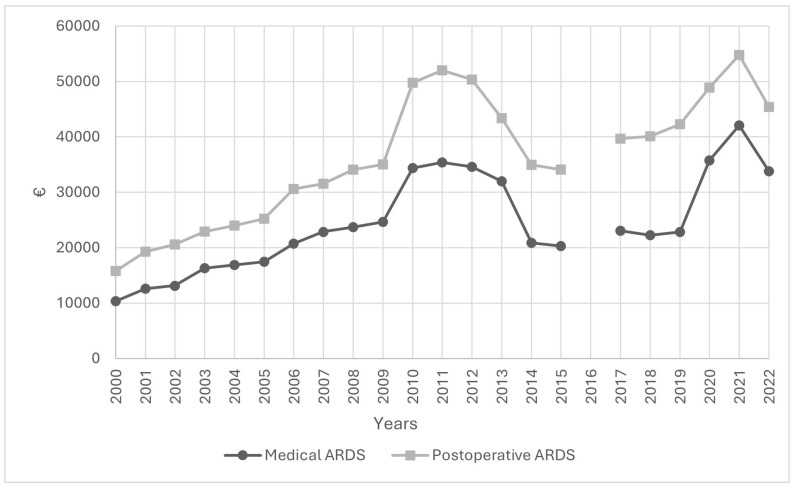
Evolution of the mean cost per medical ARDS and postoperative ARDS patient between 2000 and 2022 in Spain.

The mean healthcare costs per ARDS patient between 2000 and 2022 are shown in Figure 5. Over the course of the study period, the mean cost for postoperative ARDS patients was about 1.5 times higher than for medical patients. Overall costs increased three-fold over 22 years, while the cost for medical ARDS patients increased from EUR 10,353 in 2000 to EUR 33,810 in 2022, whereas in postoperative patients it increased from EUR 15,792 in 2000 to EUR 45,436 in 2022.

### 3.5. Effect of COVID-19 Pandemic

There was an increase in medical ARDS cases over total ARDS cases (Figure 2). In 2021, there was a decline in medical ARDS hospital mortality to 37.8% and 38.6% in postoperative ARDS hospital mortality, while in 2022 hospital mortality reached rates prior to the pandemic period (Figure 4). Healthcare costs increased during COVID-19 pandemic, peaking in 2021 with EUR 42,030 per medical ARDS patient and EUR 54,787 per postoperative ARDS patient (Figure 5).

## 4. Discussion

The major findings of our study are the following: (i) the proportion of postoperative ARDS was about 45% of total ARDS (except during the COVID-19 pandemic), with a prevalence (percentage from total surgeries) that varied between 0.05% and 0.22%; (ii) hospital mortality for postoperative ARDS was lower than for medical ARDS during the first period of the study, but it converged with medical ARDS; (iii) healthcare costs for postoperative ARDS were about 1.5 times higher than medical ARDS, aligning with an LoS 1.5 times longer; (iv) during the COVID-19 pandemic, healthcare costs reached their peak, although hospital mortality declined; and (v) gastrointestinal infection emerged as the strongest factor associated with postoperative ARDS.

Giannakoulis et al. [7] reported that ARDS is more common in males and that postoperative ARDS patients are older. However, in the second period, there was no age difference between postoperative and medical ARDS. Those authors also observed that postoperative ARDS patients were more likely to have sepsis as a risk factor and cancer as a comorbidity, as we also found in our study. Abdominal infection was common in postoperative ARDS compared with postsurgical patients without ARDS [26]. The occurrence of aspergillosis increased in our study, likely due to its prevalence as a co-infection during the COVID-19 pandemic [27], as obesity emerged as a frequent comorbidity in medical ARDS, which aligns with its role as a significant risk factor for severe COVID-19 infection [28].

Of note, we found no studies comparing postoperative ARDS epidemiology with medical ARDS epidemiology. As illustrated in Figure 2, postoperative ARDS has historically accounted for 45–50% of ARDS cases. However, this proportion declined to about 30% during the COVID-19 pandemic as a result of the surge in ARDS caused by SARS-CoV-2 infections [29]. The current literature on postoperative ARDS prevalence focuses on specific surgical procedures [6]; for instance, ARDS developed in 7.2% following hepatectomy [30], 0.4–20% after cardiac surgery [31], or 10% in patients with postoperative sepsis [26]. In contrast, studies assessing ARDS prevalence in a general surgical population [5] found a low rate of 0.2%, which aligns closely with our findings. However, the data available from the MBDS did not allow for a detailed assessment of the specific type of surgery performed on each patient. It is important to note that the period 2014–2018 is less representative when assessing postoperative ARDS prevalence; during those years, ARDS cases in Spain declined, largely attributed to the transition from ICD-9-CM to ICD-10-CM coding, a change that has been recognized in the epidemiological reporting of several conditions [32,33].

ARDS mortality has been widely studied, with hospital mortality ranging from 32% to 51%. From 2010, the hospital mortality rate has been about 45% [34]. At the beginning of the 21st century, hospital mortality for medical ARDS in Spain exceeded 55%, while postoperative ARDS had a lower mortality; this difference persisted for the first period of our study (2000–2015). Medical ARDS is known to have a higher 90-day mortality compared with postoperative ARDS, even after adjusting for confounders such as age, lung severity, and use of vasopressors [7]. In recent years, there has also been a notable decline in hospital mortality in both postoperative and medical ARDS patients. Apparently, this decline could coincide with the implementation of intraoperative lung-protective MV strategies [34,35]. Improvements in perioperative management might have contributed to lower postoperative ARDS mortality [7]. Avoiding excessive perioperative transfusions is known to reduce postoperative ARDS development [36], and implementation of intraoperative blood management has reduced transfusion-related complications, including postoperative ARDS [37].

In our study, the period 2014–2018 is less representative due to the lower number of ARDS cases. After 2020, the COVID-19 pandemic plays a key role in ARDS epidemiology, not only for becoming one of the main causes of ARDS [29] but because it was associated with a reduction in elective surgery [38], although this number declined with subsequent SARS-CoV-2 waves [39]. In Spain, during the COVID-19 pandemic emergency surgery decreased by 58.9% and higher morbidity was observed in patients undergoing emergency surgery [40]. Decline in both elective and emergency surgeries, coupled with an increase in comorbidities and advanced disease stages among patients undergoing surgery, could explain the convergence in mortality between medical and postoperative ARDS [41,42]. However, some reports declared no evidence of increased mortality or morbidity for surgeries during COVID-19 pandemic [43,44]. ECMO use in severe ARDS patients is known to reduce mortality [45], and it is a useful tool in post-cardiothoracic surgery [46], although its use in postoperative ARDS has not been widely studied. Most systematic reviews on ECMO in ARDS focus on medical ARDS patients [45,47]. When comparing our two study periods, ECMO use increased in both groups, accompanied by a decline in hospital mortality. However, we cannot ensure that clinicians have followed the multidisciplinary care bundles in all patients collected in the MBDS, so ARDS mortality might have been affected by this fact.

When evaluating the healthcare costs associated with ARDS patients, we found that the costs for postoperative ARDS were consistently about 1.5 times higher than those for medical ARDS patients, which is consistent with findings that the LoS for postoperative ARDS patients is approximately 1.5 times longer. As reported by Park et al. [48], postoperative patients tend to associate a longer ICU stay. Although we did not adjust for inflation in the cost analysis, this increase in healthcare costs cannot be fully explained by the general rise in prices in Spain, which was 68% between 2000 and 2022 [49], while ARDS healthcare costs increased by 100% to 200%. Shari et al. [50] found that compared with ARDS present at admission, hospital-acquired ARDS was more likely to develop in surgical patients and was associated with a longer adjusted hospital stay. As LoS is a well-known determinant of increased healthcare costs [51], this is particularly important given that ICU stay is three times more expensive than the stay in a general ward [52]. Early-warning systems may help reduce healthcare costs by enabling the timely identification of risk factors, which can lead to lower mortality rates and shorter hospital stays [53]. Additionally, lung-protective ventilation has demonstrated clear benefits in improving ARDS outcomes while decreasing associated costs [54].

To reduce the incidence of postoperative ARDS, as well as associated mortality and healthcare costs, integrating predictive analytics into surgical workflows to identify high-risk patients could be highly beneficial. Predictive models have already demonstrated high sensitivity and specificity in forecasting ARDS risk [55].

We acknowledge some limitations, as well as strengths, of this study. Firstly, as with any retrospective analysis, there is a possibility of under-coding variables, leading to incomplete or inaccurate information. This could introduce a potential bias and affect the robustness of our findings. Secondly, we lacked detailed data, including chest radiographs, the ratio of arterial oxygen partial pressure to fractional inspired oxygen (PaO_2_/FiO_2_), and levels of positive end-expiratory pressure (PEEP), which are essential for confirming ARDS and grading its severity. Thirdly, since MBDS data are anonymous, it is impossible to ascertain whether a patient was hospitalized more than once within the same year, across multiple years, or in different hospitals. Fourthly, the transition from the ICD-9-CM to ICD-10-CM, along with the decline in cases, makes those years less representative. Fifthly, the date of ARDS diagnosis cannot be obtained from the MBDS, as it is not possible to identify whether the ARDS diagnosis was prior to surgery. Sixthly, we acknowledge that during our study period, some changes could have occurred in the perioperative management of critically ill patients that could change the prevalence and mortality of ARDS. Moreover, due to the large sample size of this study, most *p*-values reached statistical significance. Therefore, the interpretation of the results should focus not only on statistical significance but also on the absolute differences observed among groups, on the magnitude of these differences, as well as on their clinical relevance and effect sizes, as reflected in odds ratios, means, and proportions. Although the “Present on Admission” (POA) indicator has been available in the CMBD since the implementation of the ICD-10-ES in 2016, it was not used in this study due to poor data quality and inconsistent coding patterns observed in our cohort. Following the recommendations by Walraven et al. [56], we addressed the issues posed by administrative databases to the best of our ability, acknowledging certain limitations. However, the major strength of this study is the large number of ARDS patients, which provides high statistical power and enhances the reliability of our analyses. Additionally, the long follow-up period allows for a comprehensive assessment of trends over time. Moreover, the MBDS has an estimated coverage of 99.5% of hospitals in Spain [11,12], so the vast majority of hospitals and patients are recorded in this database, giving consistency to the data. To our knowledge, this is the largest nationwide epidemiological study of ARDS with the longest available follow-up period, offering a clear view of the disease’s trend.

## 5. Conclusions

The prevalence of postoperative ARDS in the 21st century in Spain ranged between 0.05% and 0.22%, representing 45–50% of all ARDS cases. While postoperative ARDS has historically been associated with lower mortality compared with medical ARDS, recent trends indicate that these rates are converging. Postoperative ARDS incurs costs that are 1.5 times higher than those for medical ARDS, consistent with longer hospital stays. During the COVID-19 pandemic, hospital mortality rates decreased in both groups, though associated costs reached their highest levels. Gastrointestinal infections emerged as the most significant factor associated with postoperative ARDS.

## Figures and Tables

**Table 1:** Patient characteristics during the first period of the study (2000–2015).

	Medical ARDS (n = 36,393)	Postoperative ARDS (n = 31,820)	*p*-Value
**Characteristics**			
Sex (male) [% (n)]	65.1% (n = 23,700)	65.0% (n = 20,692)	**0.045**
Age (years) [mean (SD)]	56.9 (20.8)	60.3 (19.7)	**<0.001**
Charlson Index [mean (SD)]	0.7 (0.9)	0.9 (1.0)	**<0.001**
**Comorbidities [% (n)]**			
Diabetes mellitus	10.3% (n = 3747)	10.0% (n = 3166)	0.138
Obesity	4.9% (n = 1768)	4.9% (1545)	1
Chronic respiratory diseases	12.6% (n = 4598)	11.9% (3777)	**0.002**
Arterial hypertension	19.4% (n = 7046)	21.5% (6834)	**<0.001**
Ischemic heart diseases	5.7% (n = 2057)	5.7% (1814)	0.797
Cancer	14.6% (n = 5305)	22.5% (7160)	**<0.001**
HIV	1.9% (n = 685)	0.5% (164)	**<0.001**
Hepatic diseases	4.7% (n = 1695)	3.4% (1082)	**<0.001**
Renal diseases	10.6% (n = 3874)	19.2% (6089)	**<0.001**
Systemic candidiasis	1.0% (n = 361)	1.6% (492)	**<0.001**
General candidiasis	6.9% (n = 2527)	7.9% (2517)	**<0.001**
Aspergillosis	1.1% (n = 401)	0.6% (202)	**<0.001**
Influenza	2.3% (n = 819)	0.6% (179)	**<0.001**
**Sites of infection [% (n)]**			
Central nervous system	1.2% (n = 439)	1.0% (314)	**0.006**
Circulatory	0.6% (n = 210)	0.9% (294)	**<0.001**
Digestive	9.9% (n = 3618)	18.1% (5753)	**<0.001**
Genitourinary	9.0% (n = 3284)	8.6% (2732)	**0.045**
Respiratory	46.0% (n = 16,737)	37.4% (11,897)	**<0.001**
Skin	2.1% (747)	3.5% (1127)	**<0.001**
Others	14.6% (5296)	25.2% (8026)	**<0.001**
**Outcomes**			
LoS (days) [mean (SD)]	29.7 (36.02)	42.7 (45.83)	**<0.001**
Death [% (n)]	49.9% (18,168)	47.0% (14,943)	**<0.001**
Sepsis [% (n)]	66.2% (24,091)	66.0% (21,006)	0.622
ECMO [% (n)]	0.2% (63)	0.4% (116)	**<0.001**

Continuous variables are represented as the mean and standard deviation (SD); categorical variables are represented as the percentage (%) and number (n). *p* values in bold indicate statistical significance. ARDS: acute respiratory distress syndrome; ECMO: extracorporeal membrane oxygenation; HIV: human immunodeficiency virus; LoS: length of stay.

**Table 2:** Patient characteristics during the first period of the study (2017–2022).

	Medical ARDS (n = 16,198)	Postoperative ARDS (n = 8781)	*p*-Value
**Characteristics**			
Sex (male) [% (n)]	68.8% (n = 11,138)	67.5% (n = 5926)	**0.048**
Age (years) [mean (SD)]	60.4 (14.26)	60.0 (15.86)	0.061
Charlson Index [mean (SD)]	1.0 (1.4)	1.3 (1.8)	**<0.001**
**Comorbidities [% (n)]**			
Diabetes mellitus	22.5% (n = 3649)	18.3% (n = 1605)	**<0.001**
Obesity	20.7% (n = 3354)	15.0% (n = 1314)	**<0.001**
Chronic respiratory diseases	13.8% (n = 2241)	12.3% (n = 1082)	**<0.001**
Arterial hypertension	35.5% (n = 5749)	28.4% (n = 2490)	**<0.001**
Ischemic heart diseases	1.4% (n = 226)	2.8% (n = 248)	**<0.001**
Cancer	6.8% (n = 1103)	12.2% (n = 1075)	**<0.001**
HIV	0.9% (n = 146)	0.5% (n = 42)	**<0.001**
Hepatic diseases	9.5% (n = 1530)	9.4% (n = 828)	0.945
Renal diseases	6.7% (n = 1077)	6.8% (n = 595)	0.721
Systemic candidiasis	1.4% (n = 229)	2.7% (n = 239)	**<0.001**
General candidiasis	12.1% (n = 1952)	15.4% (n = 1350)	**<0.001**
Aspergillosis	4.3% (n = 695)	3.7% (n = 327)	**0.033**
Influenza	3.2% (n = 517)	2.5% (n = 215)	**<0.001**
**Sites of infection [% (n)]**			
Central nervous system	0.2% (n = 29)	0.4% (n = 37)	**<0.001**
Circulatory	0.6% (n = 93)	1.1% (n = 97)	**<0.001**
Digestive	1.6% (n = 253)	12.3% (n = 1080)	**<0.001**
Genitourinary	9.0% (n = 3284)	8.6% (n = 2732)	**0.045**
Respiratory	85.3% (n = 13,822)	65.8% (n = 5775)	**<0.001**
Skin	1.0% (n = 166)	3.1% (n = 269)	**<0.001**
Others	15.3% (n = 2485)	22.0% (n = 1934)	**<0.001**
**Outcomes**			
LoS (days) [mean (SD)]	33.3 (29.0)	45.6 (39.7)	**<0.001**
Death [% (n)]	43.2% (n = 7004)	42.7% (n = 3749)	0.413
Sepsis [% (n)]	48.9% (n = 7917)	59.1% (n = 5186)	**<0.001**
ECMO [% (n)]	2.2% (n = 350)	3.8% (n = 335)	**<0.001**

Continuous variables are represented as the mean and standard deviation (SD); categorical variables are represented as the percentage (%) and number (n). *p* values in bold indicate statistical significance. ARDS: acute respiratory distress syndrome; ECMO: extracorporeal membrane oxygenation; HIV: human immunodeficiency virus; LoS: length of stay.

## Data Availability

The datasets used and/or analyzed during the current study are available from the corresponding author upon reasonable request.

## Supplementary Materials

The following supporting information can be downloaded at: https://www.mdpi.com/article/10.3390/jcm14145125/s1, Table S1: International Classification of Diseases, 9th Revision, Clinical Modification (ICD-9-CM) codes for sepsis diagnosis; Table S1: International Classification of Diseases, 10th Revision, Clinical Modification (ICD-10-CM) codes for sepsis diagnosis; Table S3: International Classification of Diseases, 9th Revision, Clinical Modification (ICD-9-CM) codes for the site of infection; Table S4: International Classification of Diseases, 10th Revision, Clinical Modification (ICD-10-CM) codes for the site of infection; Table S5: International Classification of Diseases, 9th Revision, Clinical Modification (ICD-9-CM) codes for acute organ dysfunction; Table S6: International Classification of Diseases, 10th Revision, Clinical Modification (ICD-10-CM) codes for acute organ dysfunction; Table S7: International Classification of Diseases, 10th Revision, Clinical Modification (ICD-9-CM) codes for Charlson Index calculation; Table S8: International Classification of Diseases, 10th Revision, Clinical Modification (ICD-10-CM) codes for Charlson Index calculation; Table S9: Univariate logistic regression of the first period of the study (2000–2015); Table S10: Univariate logistic regression of the first period of the study (2017–2022).

## Abbreviations

The following abbreviations are used in this manuscript:

ARDS	acute respiratory distress syndrome
DRG	diagnosis-related groups
ECMO	extracorporeal membrane oxygenation
ICD	international classification of diseases
ICU	intensive care unit
LoS	length of hospital stay
MBDS	minimum basic dataset
MV	mechanical ventilation
PEEP	positive end-expiratory pressure
PARF	postoperative acute respiratory failure
PPCs	postoperative pulmonary complications
SD	standard deviation
