# Supplementary material for: Prevalence, Outcomes and Healthcare Costs of Postoperative ARDS Compared with Medical ARDS
Source: J Clin Med. 2025 Jul 18;14(14):5125. doi: 10.3390/jcm14145125 (PMC12295955; doi:10.3390/jcm14145125)
Supplement: Supplementary file 1 [file jcm-14-05125-s001.zip › jcm-3698072-supplementary.pdf]

Supplementary table S1: International Classification of Diseases, 9th Revision, Clinical Modification (ICD-9-CM) codes for sepsis diagnosis.

Organ System	ICD-9-CM Code	ICD-9-CM Code Description
**Nervous**		
	013.*	Tuberculosis of meninges and central nervous system
	036.*	Meningococcal infection
	091.81	Acute syphilitic meningitis (secondary)
	098.82	Neurosyphilis
	320.*	Bacterial meningitis
	321.0	Cryptococcal meningitis
	321.1	Meningitis in other fungal diseases
	324.*	Central nervous system abscess
	325	Phlebitis of intracranial sinus
	360.0	Purulent endophthalmitis
	376.0	Acute inflammation of orbit
	380.14	Malignant otitis externa
	383.0.*	Acute mastoiditis
**Circulatory**		
	093.*	Cardiovascular syphilis
	098.83 098.84	Gonococcal infections
	036.4.*	Meningococcal carditis
	391.2	Acute rheumatic myocarditis
	420.99	Acute pericarditis due to other specified organisms
	421.*	Acute or subacute endocarditis
**Respiratory**		
	010.1.*	Tuberculous pleurisy in primary progressive tuberculosis
	011.*	Pulmonary tuberculosis
	012.*	Other respiratory tuberculosis
	018.*	Miliary tuberculosis
	031.0	Pulmonary diseases due to other mycobacteria
	032.*	Diphtheria
	034.*	Streptococcal throat/scarlet fever
	098.6	Gonococcal infection of pharynx
	112.4	Candidiasis, of lung
	114.0	Primary coccidioidomycosis (pulmonary)
	114.1	Primary extrapulmonary coccidioidomycosis
	115.15	Histoplasma duboisii pneumonia
	115.05	Histoplasma capsulatum pneumonia
	115.95	Histoplasmosis pneumonia unspecified
	117.5	Cryptococcus neoformans
	117.3	Aspergillosis
	136.3	Pneumocystosis
	461.*	Acute sinusitis
	462	Acute pharyngitis
	463	Acute tonsillitis
	464.*	Acute laryngitis/tracheitis
	465.*	Acute upper respiratory infection of multiple sites/not

		otherwise specified
	475	Peritonsillar abscess
	480.*	Viral pneumonia
	481	Pneumococcal pneumonia
	482.*	Other bacterial pneumonia
	483.*	Pneumonia due to other specified organism
	485	Bronchopneumonia with organism not otherwise specified
	486	Pneumonia, organism not otherwise specified
	487.0	Influenza with pneumonia
	487.1	Influenza with other respiratory manifestations
	491.21	Acute exacerbation of obstructive chronic bronchitis
	494.*	Bronchiectasis
	510.*	Empyema
	513.*	Abscess of lung and mediastinum
**Digestive**		
	001.*	Cholera
	002.*	Typhoid/paratyphoid fever
	003.*	Other salmonella infection
	004.*	Shigellosis
	005.*	Other food poisoning
	008.0.*	Intestinal infections due to *Escherichia coli*
	008.1	Intestinal infections due to Arizona group of paracolon bacillus
	008.2	Intestinal infections due to *Aerobacter aerogenes*
	008.3	Intestinal infections due to *Proteus* (*Mirabilis morganii*)
	008.4.*	Intestinal infections due to unspecified bacteria
	008.5	Bacterial enteritis, unspecified
	009.*	Ill-defined intestinal infection
	014.*	Tuberculosis of intestines peritoneum and mesenteric glands
	129	Intestinal parasitism unspecified
	522.5	Periapical abscess without sinus
	522.7	Periapical abscess with sinus
	526.4	Inflammatory conditions of the jaw
	527.3	Abscess of the salivary glands
	528.3	Cellulitis and abscess of oral soft tissue
	540.*	Acute appendicitis
	541	Appendicitis not otherwise specified
	542	Other appendicitis
	562.01	Diverticulitis of the small intestine without hemorrhage
	562.03	Diverticulitis of the small intestine with hemorrhage
	562.11	Diverticulitis of colon without hemorrhage
	562.13	Diverticulitis of colon with hemorrhage
	566	Abscess of the anal and rectal regions
	567.*	Peritonitis
	569.5	Intestinal abscess

	569.61	Infection of colostomy or enterostomy
	569.83	Perforation of intestine
	572.0	Abscess of liver
	572.1	Portal pyemia
	575.0	Acute cholecystitis
**Genitourinary**		
	016.*	Tuberculosis of genitourinary system
	098.17	Gonococcal salpingitis specified as acute
	112.2	Candidiasis of other urogenital sites
	590.*	Kidney infection
	599.0	Urinary tract infection not otherwise specified
	601.*	Prostatic inflammation
	604.*	Orchitis and epididymitis
	614.*	Female pelvic inflammation disease
	615.*	Uterine inflammatory disease
	616.3	Abscess of Bartholin's gland
	616.4	Other abscess of vulva
**Pregnancy**		
	634.0	Spontaneous abortion, complicated by genital tract and pelvic infection
		infection
	635.0	Legally induced abortion, complicated by genital tract and
		pelvic infection
	636.0	Illegally induced abortion, complicated by genital tract and
		pelvic infection
	637.0	Unspecified abortion, complicated by genital tract and pelvic
		infection
	638.0	Failed attempted abortion, complicated by genital tract and
		pelvic infection
	639.0	Complications following abortion and ectopic and molar
		pregnancies infection
	646.6.*	Infections of genitourinary tract in pregnancy
	658.4.*	Infection of amniotic cavity
	670.*	Major puerperal infection
	675.1.*	Abscess of breast
**Skin, soft tissue, or bone**		
	003.24	Salmonella osteomyelitis
	015.*	Tuberculosis of bones and joints
	017.*	Tuberculosis of other organs
	031.1	Cutaneous diseases due to other mycobacteria
	035	Erysipelas
	036.82	Meningococcal arthropathy
	040.0	Gas gangrene
	095.5	Syphilis of bone
	098.5.*	Gonococcal infection of joint

	681.*	Cellulitis, finger/toe
	682.*	Other cellulitis or abscess
	683	Acute lymphadenitis
	685.0	Pilonidal cyst, with abscess
	686.*	Other local skin infection
	711.0	Pyogenic arthritis
	728.86	Necrotizing fasciitis
	730.*	Osteomyelitis
**Other**		
	790.7	Bacteremia
	958.3	Posttraumatic wound infection, not elsewhere classified
	996.6.*	Infection or inflammation of device/graft
	998.5.*	Postoperative infection
	999.3.*	Infectious complication of medical care not otherwise classified

Supplementary table S2: International Classification of Diseases, 10th Revision, Clinical Modification (ICD-10-CM) codes for sepsis diagnosis.

A02.1	Salmonella septicemia
A20.7	Septicemic plague
A22.7	Septicemia due to anthrax
A39.4	Meningococcal septicemia
A39.1	Waterhouse-Friderichsen syndrome
A41.2	Staphylococcal, unspecified
A41.0	Sepsis due to Staphylococcus aureus
A41.1	Sepsis due to other specified staphylococci
A40.*	Streptococcal sepsis
A41.4	Sepsis due to anaerobes
A41.50	Sepsis due to other gram-negative organisms NEOM
A41.59	Sepsis due others Gram negatives
A41.3	Sepsis due to Haemophilus influenzae
A41.51	Escherichia coli sepsis [E. coli]
A41.52	Pseudomonas sepsis
A41.53	Sepsis due to Serratia
A41.59	Sepsis due to other gram negatives
A41.89	Other specified sepsis
A41.9	Sepsis, unspecified organism (septicemia NEOM)
A54.86	Gonococcal septicemia
A48.3	Toxic shock syndrome
B37.7	Candida sepsis
B37.6	Candidal endocarditis
B38.7	Disseminated coccidioidomycosis
N39.0<br>B37.4.*<br>N30.*<br>N34.*<br>R82.81	Urinary tract infection
R65.*	Symptoms and signs specifically associated with systemic inflammation and infection
R78.81	Bacteremia
T80.89	Complication of medical care, other transfusion reaction
T81.1.*	Postprocedural shock
T81.4.*	Infection following a procedure
R65.21	Shock septic
B37.5	Candidal meningitis
B37.6	Candidal endocarditis
B37.7	Candidal sepsis
B37.8	Candidiasis of other sites
B37.9	Candidiasis, unspecified
B44.0	Invasive pulmonary aspergillosis

B44.1	Other pulmonary aspergillosis
B44.2	Tonsillar aspergillosis
B44.7	Disseminated aspergillosis
B44.8	Other forms of aspergillosis
B44.9	Aspergillosis, unspecified
B45.0	Pulmonary cryptococcosis
B45.1	Cerebral cryptococcosis
B45.2	Cutaneous cryptococcosis
B45.3	Osseous cryptococcosis
B45.7	Disseminated cryptococcosis
B45.8	Other forms of cryptococcosis
B45.9	Cryptococcosis, unspecified
B46.0	Pulmonary mucormycosis
B46.1	Rhinocerebral mucormycosis
B46.2	Gastrointestinal mucormycosis
B46.3	Cutaneous mucormycosis
B46.4	Disseminated mucormycosis
B46.5	Mucormycosis, unspecified
B46.8	Other zygomycoses
B46.9	Zygomycosis, unspecified
B49	Unspecified mycosis

Supplementary Table S3: International Classification of Diseases, 9th Revision, Clinical Modification (ICD-9-CM) codes for the site of infection.

Organ System	ICD-9-CM Code	ICD-9-CM Code Description
**Nervous**		
	013.*	Tuberculosis of meninges and central nervous system
	036.*	Meningococcal infection
	091.81	Acute syphilitic meningitis (secondary)
	098.82	Neurosyphilis
	320.*	Bacterial meningitis
	321.0	Cryptococcal meningitis
	321.1	Meningitis in other fungal diseases
	324.*	Central nervous system abscess
	325	Phlebitis of intracranial sinus
	360.0	Purulent endophthalmitis
	376.0	Acute inflammation of orbit
	380.14	Malignant otitis externa
	383.0.*	Acute mastoiditis
**Circulatory**		
	093.*	Cardiovascular syphilis
	098.83 098.84	Gonococcal infections
	036.4.*	Meningococcal carditis
	391.2	Acute rheumatic myocarditis
	420.99	Acute pericarditis due to other specified organisms
	421.*	Acute or subacute endocarditis
**Respiratory**		
	010.1.*	Tuberculous pleurisy in primary progressive tuberculosis
	011.*	Pulmonary tuberculosis
	012.*	Other respiratory tuberculosis
	018.*	Miliary tuberculosis
	031.0	Pulmonary diseases due to other mycobacteria
	032.*	Diphtheria
	034.*	Streptococcal throat/scarlet fever
	098.6	Gonococcal infection of pharynx
	112.4	Candidiasis, of lung
	114.0	Primary coccidioidomycosis (pulmonary)
	114.1	Primary extrapulmonary coccidioidomycosis
	115.15	Histoplasma duboisii pneumonia
	115.05	Histoplasma capsulatum pneumonia
	115.95	Histoplasmosis pneumonia unspecified
	117.5	Cryptococcus neoformans
	117.3	Aspergillosis
	136.3	Pneumocystosis
	461.*	Acute sinusitis
	462	Acute pharyngitis
	463	Acute tonsillitis
	464.*	Acute laryngitis/tracheitis
	465.*	Acute upper respiratory infection of multiple sites/not

		otherwise specified
	475	Peritonsillar abscess
	480.*	Viral pneumonia
	481	Pneumococcal pneumonia
	482.*	Other bacterial pneumonia
	483.*	Pneumonia due to other specified organism
	485	Bronchopneumonia with organism not otherwise specified
	486	Pneumonia, organism not otherwise specified
	487.0	Influenza with pneumonia
	487.1	Influenza with other respiratory manifestations
	491.21	Acute exacerbation of obstructive chronic bronchitis
	494.*	Bronchiectasis
	510.*	Empyema
	513.*	Abscess of lung and mediastinum
**Digestive**		
	001.*	Cholera
	002.*	Typhoid/paratyphoid fever
	003.*	Other salmonella infection
	004.*	Shigellosis
	005.*	Other food poisoning
	008.0.*	Intestinal infections due to *Escherichia coli*
	008.1	Intestinal infections due to Arizona group of paracolon bacillus
	008.2	Intestinal infections due to *Aerobacter aerogenes*
	008.3	Intestinal infections due to *Proteus* (*Mirabilis morganii*)
	008.4.*	Intestinal infections due to unspecified bacteria
	008.5	Bacterial enteritis, unspecified
	009.*	Ill-defined intestinal infection
	014.*	Tuberculosis of intestines peritoneum and mesenteric glands
	129	Intestinal parasitism unspecified
	522.5	Periapical abscess without sinus
	522.7	Periapical abscess with sinus
	526.4	Inflammatory conditions of the jaw
	527.3	Abscess of the salivary glands
	528.3	Cellulitis and abscess of oral soft tissue
	540.*	Acute appendicitis
	541	Appendicitis not otherwise specified
	542	Other appendicitis
	562.01	Diverticulitis of the small intestine without hemorrhage
	562.03	Diverticulitis of the small intestine with hemorrhage
	562.11	Diverticulitis of colon without hemorrhage
	562.13	Diverticulitis of colon with hemorrhage
	566	Abscess of the anal and rectal regions
	567.*	Peritonitis
	569.5	Intestinal abscess

	569.61	Infection of colostomy or enterostomy
	569.83	Perforation of intestine
	572.0	Abscess of liver
	572.1	Portal pyemia
	575.0	Acute cholecystitis
**Genitourinary**		
	016.*	Tuberculosis of genitourinary system
	098.17	Gonococcal salpingitis specified as acute
	112.2	Candidiasis of other urogenital sites
	590.*	Kidney infection
	599.0	Urinary tract infection not otherwise specified
	601.*	Prostatic inflammation
	604.*	Orchitis and epididymitis
	614.*	Female pelvic inflammation disease
	615.*	Uterine inflammatory disease
	616.3	Abscess of Bartholin's gland
	616.4	Other abscess of vulva
**Pregnancy**		
	634.0	Spontaneous abortion, complicated by genital tract and pelvic infection
		infection
	635.0	Legally induced abortion, complicated by genital tract and
		pelvic infection
	636.0	Illegally induced abortion, complicated by genital tract and
		pelvic infection
	637.0	Unspecified abortion, complicated by genital tract and pelvic
		infection
	638.0	Failed attempted abortion, complicated by genital tract and
		pelvic infection
	639.0	Complications following abortion and ectopic and molar
		pregnancies infection
	646.6.*	Infections of genitourinary tract in pregnancy
	658.4.*	Infection of amniotic cavity
	670.*	Major puerperal infection
	675.1.*	Abscess of breast
**Skin, soft tissue, or bone**		
	003.24	Salmonella osteomyelitis
	015.*	Tuberculosis of bones and joints
	017.*	Tuberculosis of other organs
	031.1	Cutaneous diseases due to other mycobacteria
	035	Erysipelas
	036.82	Meningococcal arthropathy
	040.0	Gas gangrene
	095.5	Syphilis of bone
	098.5.*	Gonococcal infection of joint

	681.*	Cellulitis, finger/toe
	682.*	Other cellulitis or abscess
	683	Acute lymphadenitis
	685.0	Pilonidal cyst, with abscess
	686.*	Other local skin infection
	711.0	Pyogenic arthritis
	728.86	Necrotizing fasciitis
	730.*	Osteomyelitis
**Other**		
	790.7	Bacteremia
	958.3	Posttraumatic wound infection, not elsewhere classified
	996.6.*	Infection or inflammation of device/graft
	998.5.*	Postoperative infection
	999.3.*	Infectious complication of medical care not otherwise classified

Supplementary Table S4: International Classification of Diseases, 10th Revision, Clinical Modification (ICD-10-CM) codes for the site of infection.

	**Nervous**
A17	Tuberculosis of meninges and central nervous system
A39.0	Meningococcal infection
A51.41	Acute syphilitic meningitis (secondary)
A52.1	symptomatic neurosyphilis
A52.2	Asymptomatic neurosyphilis
A52.3	Unspecified neurosyphilis
G00	Bacterial meningitis, not elsewhere classified
G04.2	Bacterial meningoencephalitis and meningomyelitis, not elsewhere classified
B45.1	Cerebral cryptococcosis
G02	Meningitis in other infectious and parasitic diseases classified elsewhere
G06	Central nervous system abscess
G08	Phlebitis of intracranial sinus
H44.0	Purulent endophthalmitis
H05.00	Acute inflammation of orbit
H60.20	Malignant otitis externa
H70.0	Acute mastoiditis
	**Circulatory**
A52.00-A52.04	Cardiovascular syphilis
A52.06	Cardiovascular syphilis
A52.09	Cardiovascular syphilis
A54.83	gonococcal heart infection (endocarditis, pericarditis, myocarditis)
I01.2	Acute rheumatic myocarditis
I30	Acute pericarditis
I33	Acute or subacute endocarditis
	**Respiratory**
A15	respiratory tuberculosis
A31.0	Pulmonary diseases due to other mycobacteria
A36	Diphtheria
A38	Streptococcal throat/scarlet fever
A54.5	Gonococcal infection of pharynx
B37.1	Candidiasis, of lung
B38.0	Acute pulmonary coccidioidomycosis
B38.1	Chronic pulmonary coccidioidomycosis
B39.5	Histoplasma duboisii pneumonia
B39.2	Histoplasma capsulatum pneumonia
B39.9	Histoplasmosis pneumonia unspecified
B45	Cryptococcus neoformans
B44	Aspergillosis

B59	Pneumocystosis
J01	Acute sinusitis
J02	Acute pharyngitis
J03	Acute tonsillitis
J04	Acute laryngitis/tracheitis
J06	Acute upper respiratory infection of multiple sites/not
J36	Peritonsillar abscess
J12	Viral pneumonia
J13	Pneumococcal pneumonia
J15	Other bacterial pneumonia
J16	Pneumonia due to another specified organism
J18	Pneumonia, organism not otherwise specified
J10	Influenza
J44.1	Acute exacerbation of obstructive chronic bronchitis
J47	Bronchiectasis
J86	Pyothorax
J85	Abscess of lung and mediastinum
	**Digestive**
A00	Cholera
A01	Typhoid/paratyphoid fever
A02	Other salmonella infection
A03	Shigellosis
A05	Other food poisoning
A04.0-A04.4	Intestinal infections due to *Escherichia coli*
A04.8	Other specified bacterial intestinal infections
A04.9	Bacterial intestinal infection, unspecified
A18.3	Tuberculosis of intestines peritoneum and mesenteric glands
B82	Intestinal parasitism unspecified
K04.7	Periapical abscess without sinus
K04.6	Periapical abscess with sinus
M27.2	Inflammatory conditions of the jaw
K11.3	Abscess of the salivary glands
K12.2	Cellulitis and abscess of oral soft tissue
K35	Acute appendicitis
K37	Appendicitis not otherwise specified
K36	Other appendicitis
K57.12	Diverticulitis of the small intestine without haemorrhage
K57.13	Diverticulitis of the small intestine with haemorrhage
K57.32	Diverticulitis of colon without haemorrhage
K57.33	Diverticulitis of colon with haemorrhage
K61.0	Anal abscess
K61.1	rectal abscess
K61.3	Ischiorectal abscess

K65	Peritonitis
K63.0	Intestinal abscess
K94.02	Infection of colostomy
K94.12	Infection of enterostomy
K63.1	Perforation of intestine
K75.0	Abscess of liver
K75.1	Portal pyaemia
K81.0	Acute cholecystitis
	**Genitourinary**
A18.1	Tuberculosis of genitourinary system
A54.24	Gonococcal salpingitis specified as acute
B37.4	Candidiasis of other urogenital sites
N10	Kidney infection
N39.0	Urinary tract infection not otherwise specified
N41	Prostatic inflammation
N45	Orchitis and epididymitis
N73	Female pelvic inflammation disease
N71	Uterine inflammatory disease
N75.1	Abscess of Bartholin's gland
N76.4	Other abscess of vulva
	**Pregnancy**
O03	Spontaneous abortion
O04	Complications after (induced) termination of pregnancy
O08	Complications following abortion and ectopic and molar pregnancies
O23	Infections of genitourinary tract in pregnancy
O41.1	Infection of amniotic cavity
O85	Major puerperal infection
N61.1	Abscess of breast
	**Skin, soft tissue, or bone**
A02.24	Salmonella osteomyelitis
A18.0	Tuberculosis of bones and joints
A18	Tuberculosis of other organs
A31.1	Cutaneous diseases due to other mycobacteria
A146	Erysipelas
A39.83	Meningococcal arthropathy
A48.0	Gas gangrene
A52.77	Syphilis of bone
A54.5	Gonococcal infection of joint
L03	Cellulitis and acute lymphangitis
L05.01	Pilonidal cyst, with abscess
L08	Other local skin infection
M00	Pyogenic arthritis

M72.6	Necrotizing fasciitis
M86	Osteomyelitis
	**Other**
R78.81	Bacteraemia
T79.8XXA	Post-traumatic wound infection, not elsewhere classified
T82.7	Infection or inflammation of device/graft
T81. 4	Postoperative infection

Supplementary Table S5: International Classification of Diseases, 9th Revision, Clinical Modification (ICD-9-CM) codes for acute organ dysfunction.

Organ System	ICD-9-CM Code	ICD-9-CM Code Description
Cardiovascular	427.5	Cardiac arrest
	458.0	Orthostatic hypotension
	458.8	Other specified hypotension
	458.9	Hypotension, unspecified
	785.5.*	Shock without mention of trauma
	796.3	Hypotension, transient
Hematologic	286.2	Disseminated intravascular coagulation
	286.6	Defibrination syndrome
	286.9	Other and unspecified coagulation defects
	287.3.*<br>287.4.*<br>287.5	Thrombocytopenia, primary, secondary or unspecified
	790.92	Abnormal coagulation profile
Hepatic	570	Acute and subacute necrosis of liver
	572.2	Hepatic encephalopathy
	573.3	Hepatitis (septic & not elsewhere classified)
	573.4	Hepatic infarction
Neurologic	293.*	Transient organic psychosis
	348.1	Anoxic brain damage
	348.3.*	Encephalopathy, acute
	780.01	Coma
	780.09	Altered consciousness, unspecified
	89.14	Electroencephalography
Renal	580.*	Acute glomerulonephritis
	584.*	Acute renal failure
	586	Renal shutdown, renal failure unspecified
	39.95	Hemodialysis
Respiratory	518.5.*	Pulmonary insufficiency following trauma and surgery
	518.8.*	Respiratory failure
	786.03	Apnea
	799.1	Respiratory arrest
	786.09	Respiratory insufficiency
	96.7.*	Ventilator management
	96.04	Endotracheal intubation (emergency procedure)
	93.90	Continuous positive airway pressure^{o}
Metabolic	276.2	Acidosis, metabolic or lactic

Supplementary Table S6: International Classification of Diseases, 10th Revision, Clinical Modification (ICD-10-CM) codes for acute organ dysfunction.

Organ System	ICD-10-CM Code	ICD-10-CM Code Description
**Cardiovascular**	I46	Cardiac arrest
	I95.1	Orthostatic hypotension
	I95.89	Other specified hypotension
	I95.9	Hypotension, unspecified
	R57.0	Shock without mention of trauma
	R57.9	Hypotension, transient
**Hematologic**	D65	Disseminated intravascular coagulation (Defibrination syndrome)
	D68	Other and unspecified coagulation defects
	D69	Purpura and other bleeding conditions
	R79.1	Abnormal coagulation profile
**Hepatic**	K72.0	Acute and subacute necrosis of liver
	K72.01, K72.91	Hepatic encephalopathy
	K75.9	Hepatitis (septic & not elsewhere classified)
	K76.3	Hepatic infarction
**Neurologic**	F06	Transient organic psychosis
	G93.1	Anoxic brain damage
	G93.4	Other and unspecified types of encephalopathy
	G31.2	Alcoholic encephalopathy
	G94	Other disorders of the brain in diseases classified elsewhere
	I67.4	Hypertensive encephalopathy
	R40	Drowsiness, stupor and coma
	4A00	Electroencephalography
**Renal**	N00	Acute glomerulonephritis
	N17	Acute renal failure
	N19	Renal shutdown, renal failure unspecified
	5A1D	Hemodialysis
**Respiratory**	J95	Intraoperative and postprocedural complications and disorders of the respiratory system, not elsewhere classified
	R06.81	Apnea
	R09.2	Respiratory arrest
	J96	Respiratory insufficiency
	5A19, 5A09	Ventilator management
	0BH1	Endotracheal intubation (emergency procedure)
	A09357, 5A09557, 5A09457	Continuous positive airway pressure
**Metabolic**	E87.2	Acidosis, metabolic or lactic

Supplementary table S7. International Classification of Diseases, 10th Revision, Clinical Modification (ICD-9-CM) codes for Charlson Index calculation.

**Comorbidities**	**ICD-9-CM**
Myocardial infarction	410.*, 412.*
Congestive heart failure	428.*
Peripheral vascular disease	443.9, 441.*, 785.4, V43.4, Procedure 38.48
Cerebrovascular disease	430.*, 431.*,432, 433.*, 434.*, 435.*, 436.*, 437.*, 438.*
Dementia	290.*
Chronic pulmonary disease	490.*–505.x*, 506.4
Rheumatic disease	710.0, 710.1, 710.4, 714.0–714.2, 714.81, 725.x
Peptic ulcer disease	531.*–534.*
Mild liver disease	571.2, 571.4.*, 571.5, 571.6
Diabetes without chronic complication	250.0.*, 250.1.*, 250.2.*, 250.3.*, 250.7.*
Diabetes with chronic complication	250.4.*, 240.5.*, 250.6.*
Hemiplegia or paraplegia	344.1, 342.*
Renal disease	582.*, 583.0, 583.1, 583.2, 583.4, 583.6, 583.7, 585.*, 586.*, 588.*
Any malignancy, including lymphoma and leukemia, except malignant neoplasm of skin	140.*–172.*, 174.*–195.8, 200.*–208.*
Moderate or severe liver disease	456.0–456.21, 572.2–572.8
Metastatic solid tumor	196.*–199.1
AIDS/HIV	042.*

Supplementary table S8. International Classification of Diseases, 10th Revision, Clinical Modification (ICD-10-CM) codes for Charlson Index calculation.

Myocardial infarction	I21.*<br>I22.*<br>I25.2
Congestive heart failure	I09.9<br>I11.0<br>I13.0<br>I13.2<br>I25.5<br>I42.0<br>I42.5<br>I42.6<br>I42.7<br>I42.8<br>I42.9<br>I43<br>I50.*
Peripheral vascular disease	I70.*<br>I71.*<br>I67.1<br>I73.1<br>I73.8.*<br>I73.9<br>I77.1<br>I79.0<br>K55.1<br>K55.8<br>K55.9<br>Z95.8.*<br>Z95.9
Cerebrovascular disease	G45,*<br>G46.*<br>H34.*<br>I60.*<br>I61.*<br>I62.*<br>I63.*<br>I65.*<br>I66.*<br>I67.*<br>I68.*<br>I69.*
Dementia	F01.*<br>F02.*<br>F03.*<br>G30.*<br>G31.1
Chronic pulmonary disease	I27.8<br>I27.9

	J40.*<br>J41.*<br>J42.*<br>J43.*<br>J44.*<br>J45.*<br>J47.*<br>J60.*<br>J61.*<br>J62.*<br>J63.*<br>J64.*<br>J65.*<br>J66.*<br>J67.*<br>J68.4<br>J70.1<br>J70.3
Rheumatic disease	M05.*<br>M06.*<br>M31.5<br>M32.*<br>M33.*<br>M34.*<br>M35.1<br>M35.3<br>M36.0
Peptic ulcer disease	K25.*<br>K26.*<br>K27.*<br>K28.*
Mild liver disease	B18.*<br>K70.0<br>K70.1.*<br>K70.2<br>K70.3.*<br>K70.9<br>K71.3<br>K71.4<br>K71.5.*<br>K71.7<br>K73.*<br>K74.*<br>K76.0<br>K76.2<br>K76.3<br>K76.4<br>K76.8.*<br>K76.9<br>Z94.4
Diabetes without chronic complication	E10.1.*

	E10.6.*<br>E10.9<br>E11.0.*<br>E11.1.*<br>E11.6.*<br>E11.8<br>E11.9<br>E13.0.*<br>E13.1.*<br>E13.6.*<br>E13.8<br>E13.9
Diabetes with chronic complication	E10.2.*<br>E10.3.*<br>E10.4.*<br>E10.5.*<br>E11.2.*<br>E11.3.*<br>E11.4.*<br>E11.5.*<br>E13.2.*<br>E13.3.*<br>E13.4.*<br>E13.5.*
Paraplegia and hemiplegia	G80.*<br>G81.*<br>G82.*<br>G04.1<br>G11.4<br>G83.0<br>G83.1.*<br>G83.2.*<br>G83.3.*<br>G83.4<br>G83.9.*
Renal disease	N18.*<br>N19.*<br>N05.2<br>N05.3<br>N05.4<br>N05.5<br>N05.6<br>N05.7<br>N25.0<br>I12.0<br>I13.1.*<br>N03.2<br>N03.3<br>N03.4<br>N03.5<br>N03.6

	N03.7<br>Z49.*<br>Z94.0<br>Z99.2
Any malignancy, including lymphoma and leukaemia, except malignant neoplasm of skin	C00.*-C26.*<br>C30.*-C34.*<br>C37.*-C41.*<br>C43.*<br>C45.*-C58.*<br>C60.*-C76.*<br>C81.*-C85.*<br>C88.*<br>C90.*-C97.*
Moderate or severe liver disease	K70.4<br>K71.1.*<br>K72.1.*<br>K72.9.*<br>K76.5<br>K76.6<br>K76.7<br>I85.0.*<br>I86.4<br>I98.2
Metastatic carcinoma	C77.*<br>C78.*<br>C79.*<br>C80.*
AIDS/HIV	B20.*

Supplementary table S9. Univariate logistic regression of the first period of the study (2000-2015).

	OR	95% IC	p-value
**Charlson Index**	1.2110	(1.1904, 1.2318)	<0.0001
**Diabetes mellitus**	0.8816	(0.8381, 0.9273)	<0.0001
**Obesity**	0.9837	(0.9168, 1.0554)	0.64691
**Chronic respiratory diseases**	0.8621	(0.8227, 0.9033)	<0.0001
**Arterial hypertension**	1.0328	(0.9937, 1.0734)	0.10155
**Ischemic heart diseases**	0.9238	(0.8650, 0.9864)	0.01791
**HIV**	0.3057	(0.2567, 0.3619)	<0.0001
**Hepatic diseases**	0.7365	(0.6811, 0.7961)	<0.0001
**Renal diseases**	1.8843	(1.8034, 1.9691)	<0.0001
**Systemic candidiasis**	1.5894	(1.3863, 1.8240)	<0.0001
**General candidiasis**	1.1558	(1.0911, 1.2243)	<0.0001
**Aspergillosis**	0.5767	(0.4857, 0.6827)	<0.0001
**Central nervous system**	0.9306	(0.8030, 1.0771)	0.33652
**Circulatory**	1.5870	(1.3290, 1.8984)	<0.0001
**Digestive**	1.9221	(1.8377, 2.0106)	<0.0001
**Genitourinary**	0.9419	(0.8928, 0.9936)	0.02827
**Respiratory**	0.7028	(0.6815, 0.7248)	<0.0001
**Skin**	1.7480	(1.5916, 1.9208)	<0.0001
**Others**	1.9561	(1.8819, 2.0334)	<0.0001
**Sepsis**	0.9796	(0.9489, 1.0114)	0.20657
**ECMO**	2.6162	(1.9276, 3.5835)	<0.0001

Supplementary table S10. Univariate logistic regression of the second period of the study (2017-2022).

	OR	95% IC	p- value
**Charlson Index**	1.1069	(1.0888, 1.1253)	<0.0001
**Diabetes mellitus**	0.7726	(0.7230, 0.8253)	<0.0001
**Obesity**	0.6633	(0.6181, 0.7114)	<0.0001
**Chronic respiratory diseases**	0.8810	(0.8147, 0.9522)	<0.0001
**Arterial hypertension**	0.7171	(0.6767, 0.7597)	<0.0001
**Ischemic heart diseases**	2.0904	(1.7420, 2.5099)	<0.0001
**Cancer**	1.9176	(1.7551, 2.0950)	<0.0001
**HIV**	0.5189	(0.3633, 0.7254)	0.00019
**Hepatic diseases**	0.9968	(0.9117, 1.0891)	0.94388
**Renal diseases**	1.0355	(0.9323, 1.1491)	0.51331
**Systemic candidiasis**	1.9567	(1.6291, 2.3506)	<0.0001
**General candidiasis**	1.3261	(1.2302, 1.4291)	<0.0001
**Aspergillosis**	0.8669	(0.7572, 0.9905)	0.03697
**Influenza**	0.7463	(0.6337, 0.8760)	<0.0001
**Central nervous system**	2.3358	(1.4387, 3.8287)	0.00064
**Circulatory**	1.9357	(1.4544, 2.5778)	<0.0001
**Digestive**	8.8701	(7.7279, 10.2192)	<0.0001
**Genitourinary**	0.9325	(0.8732, 0.9955)	0.03649
**Respiratory**	0.3289	(0.3090, 0.3500)	<0.0001
**Skin**	3.0468	(2.5096, 3.7097)	<0.0001
**Others**	1.5640	(1.4638, 1.6710)	<0.0001
**Sepsis**	1.5131	(1.4356, 1.5949)	<0.0001
**ECMO**	1.7861	(1.5318, 2.0823)	<0.0001
